# Magnetic resonance guided SBRT reirradiation in locally recurrent prostate cancer: a multicentric retrospective analysis

**DOI:** 10.1186/s13014-023-02271-y

**Published:** 2023-05-22

**Authors:** Luca Boldrini, Angela Romano, Giuditta Chiloiro, Stefanie Corradini, Viola De Luca, Valeria Verusio, Andrea D’Aviero, Alessandra Castelluccia, Anna Rita Alitto, Francesco Catucci, Gianmarco Grimaldi, Christian Trapp, Juliane Hörner-Rieber, Domenico Marchesano, Vincenzo Frascino, Gian Carlo Mattiucci, Vincenzo Valentini, Piercarlo Gentile, Maria Antonietta Gambacorta

**Affiliations:** 1 Dipartimento di Diagnostica per Immagini, Radioterapia Oncologica ed Ematologia, Fondazione Policlinico Universitario Agostino Gemelli IRCCS, Rome, Italy; 2 Istituto di Radiologia, Università Cattolica del Sacro Cuore, Rome, Italy; 3 Department of Radiation Oncology, University Hospital, LMU Munich, Munich, Germany; 4 Radiation Oncology, Mater Olbia Hospital, Olbia, Sassari, Italy; 5 Radiation Oncology, Ospedale San Pietro Fatebenefratelli di Roma, Rome, Italy; 6 Radiation Oncology, Department of Radiotherapy, Hospital “A. Perrino”, ASL Brindisi, Brindisi, Italy; 7 Heidelberg Ion-Beam Therapy Center (HIT), Department of Radiation Oncology, Heidelberg University Hospital, Heidelberg, Germany

**Keywords:** Prostate cancer, Radiotherapy, Reirradiation, MRgRT, SBRT

## Abstract

**Aims:**

Reirradiation of prostate cancer (PC) local recurrences represents an emerging challenge for current radiotherapy. In this context, stereotactic body radiation therapy (SBRT) allows the delivery of high doses, with curative intent. Magnetic Resonance guided Radiation Therapy (MRgRT) has shown promising results in terms of safety, feasibility and efficacy of delivering SBRT thanks to the enhanced soft tissue contrast and the online adaptive workflow. This multicentric retrospective analysis evaluates the feasibility and efficacy of PC reirradiation, using a 0.35 T hybrid MR delivery unit.

**Methods:**

Patients affected by local recurrences of PC and treated in five institutions between 2019 and 2022 were retrospectively collected. All patients had undergone previous Radiation Therapy (RT) in definitive or adjuvant setting. Re-treatment MRgSBRT was delivered with a total dose ranging from 25 to 40 Gy in 5 fractions. Toxicity according to CTCAE v 5.0 and treatment response were assessed at the end of the treatment and at follow-up.

**Results:**

Eighteen patients were included in this analysis. All patients had previously undergone external beam radiation therapy (EBRT) up to a total dose of 59.36 to 80 Gy. Median cumulative biologically effective dose (BED) of SBRT re-treatment was 213,3 Gy (103,1-560), considering an α/β of 1.5. Complete response was achieved in 4 patients (22.2%). No grade ≥ 2 acute genitourinary (GU) toxicity events were recorded, while gastrointestinal (GI) acute toxicity events occurred in 4 patients (22.2%).

**Conclusion:**

The low rates of acute toxicity of this experience encourages considering MRgSBRT a feasibile therapeutic approach for the treatment of clinically relapsed PC. Accurate gating of target volumes, the online adaptive planning workflow and the high definition of MRI treatment images allow delivering high doses to the PTV while efficiently sparing organs at risk (OARs).

## Introduction

Prostate cancer (PC) is the most common malignancy in terms of incidence in men and currently represents the second leading cause of death [1].

Nevertheless, the possibility to early diagnose it and the recent advancements of treatment strategies are changing the framework of the management of this disease with promising outcomes [2].

The current treatment approach generally involves the use of surgery, androgen deprivation therapy (ADT) or radiation therapy (RT), either administered individually or in combination, depending on the stage of disease [3].

In particular, RT plays a well-standardized role in the radical, adjuvant or palliative treatment settings [4–7].

An emerging issue is the most appropriate management of disease in the case of a local relapse after previous radical treatment, as nearly one third of the patients experience biochemical or macroscopical relapse after primary treatment, of which 30–47% have previously undergone RT [8, 9].

The most appropriate approach in this clinical setting remains controversial also due to the limited number of evidence providing clear recommendations and consensus statements.

In this context, several treatment options have been investigated and ADT has traditionally been considered as a safe treatment modality [10]. Local treatment approaches including surgery and focal therapies have also been investigated with varying outcomes [11–15].

Considering recurrent PC reirradiation, High-Dose-Rate (HDR) Brachytherapy has historically been considered as a safe, even if minimally invasive, approach due to the possibility of delivering high doses to the target while avoiding healthy tissues [16]. Recently, Munoz et al. performed a systematic review regarding the efficacy and safety of PC reirradiation, demonstrating that this approach appears to be promising in terms of overall survival and biochemical disease control rates with no significant toxicity burden [17].

The risk of severe radiation-induced complications represents the main limiting factor for reirradiation, especially in the pelvic region with several dose-limiting organs at risk (OARs) such as bowel loops, sigmoid, bladder, urethra, rectum, cauda equina, nerves, and femoral heads [18, 19].

For this reason, it was deemed necessary to adopt delivery techniques that would allow the best achievable sparing of surrounding OARs while assuring the delivery of ablative radiation doses.

The introduction of intensity-modulated RT (IMRT) techniques has led to an increased precision in the delivery process by a rapid dose falloff, characterized by a steep dose gradient close to the target volumes. Furthermore, the implementation of stereotactic body radiation therapy (SBRT) assured the possibility to efficaciously shape the dose offering the advantage of delivering high doses even on very small target volumes or particularly close to radiosensitive OARs.

Previous experiences reported in literature demonstrate that the use of SBRT delivered with conventional linear accelerators or Cyberknife (CK; Accuray, Sunnyvale, CA, USA) is a technically feasible, effective and safe approach [20–23].

In more recent years, the introduction of hybrid magnetic resonance (MR) delivery units has made SBRT delivery feasible for lesions located in different anatomical sites, including PC [24–27]. MR-guided stereotactic RT (MRgSBRT) allows to combine the advantages of SBRT treatment with the use of MR imaging, improving visualization of the target and OARs and the possibility of performing gating and online adaptive treatment protocols [28]. The advantage of MRgSBRT in the management of prostate and prostate-bed reirradiation has recently been investigated, showing encouraging results in terms of feasibility, toxicity reduction and clinical outcomes [29, 30].

This retrospective multicentric analysis was designed in order to provide early results in terms of feasibility and effectiveness of MRgSBRT PC-reirradiation performed with a 0.35 T MRgRT unit.

## Methods

Patients affected by recurrent PC who underwent MRgSBRT reirradiation using a 0.35 T MR-linear accelerator (MRIdian, ViewRay, Mountain View, CA, USA) in five different institutions were considered for the analysis and their data were retrospective collected.

Patients were addressed to SBRT treatment following multidisciplinary discussion and signed specific informed consent to MRgSBRT treatment.

Magnetic Resonance Imaging (MRI) safety screening forms were administered to all patients before therapy.

Inclusion criteria were: age > 18 years; radiological diagnosis of recurrent PC by means of MRI and F-choline- or Ga-PSMA-PET/TC due to a rising PSA value; previous RT treatment to prostate or prostate bed.

Clinical contraindications to MRI (e.g. claustrophobia, presence of non-MRI safe devices) or specific consent deny were considered as exclusion criteria. Patients’ characteristics are summarized in Table 1.

**Table 1:** Patients characteristics

Characteristics	Patients (n = 18)
Median age (at recurrence time)	77 years (range 60–86)
Gleason grade at diagnosis	
*1* *2* *3* *4* *5*	4 (22.2%) 5 (27.8%) 3 (16.7%) 4 (22.2%) 2 (11.1%)
Prior Prostatectomy	
*Yes* *No*	9 (50%) 9 (50%)
EBRT EQD2 dose received	
*≥ 60 < 70* *≥ 70*	11 (61.1%) 7 (38.9%)
Time to relapse (years)	
*< 10* *> 10*	6 (33.3%) 12 (66.7%)
Type of relapse	
*Biochemical alone* *Macroscopic alone* *Both*	0 3 (16.7%) 15 (83.3%)
Relapse site	
*Intraprostatic/Prostate bed* *Extraprostatic* *Both*	15 (83.3%) 0 3 (16.7%)
Spacer positioning	
*Yes* *No*	2 (11.1%) 16 (88.9%)
Median PSA value pre-MRgSBRT (ng/ml)	1.38 (range 0.14–13.8)
Re-treatment BED (α/β of 1.5)	
*≤ 213.3 Gy* *> 213.3 Gy*	12 (66.7%) 6 (33.3%)
Concurrent ADT	
Yes No	10 (55.6%) 8 (44.4%)

For the simulation procedure patients were required to perform bladder preparation by drinking 500 cc of water 30 min before the scan. An enema was required 3 h before the scan to guarantee rectal emptiness.

Two patients underwent prostate-rectal spacer implantation.

The same preparation approach was maintained for each treatment fraction.

Simulation scan was performed with patients in supine position using dedicated repositioning devices.

The irradiation field definition was performed using a 25-seconds MR true fast imaging with steady-state free precession (TRUFI) sequence; while a high resolution 175-seconds TRUFI sequence was acquired for the definition of target volumes and OARs. A non-contrast-enhanced simulation CT was acquired subsequently and fused with MRI simulation imaging in order to provide electron densities for the plan calculation.

Gross tumor volume (GTV) included the entire gross tumor relapse defined by means of MRI imaging or PET/CT. The clinical target volume (CTV) was considered equal to GTV and an isotropic 3–5 mm margin was added to GTV to generate the planning target volume (PTV).

The OARs considered were: rectum, anal canal, small intestine, bladder, urethra, femoral heads and penile bulb.

The plan objectives for target optimization were to have at least 95% of the PTV to be covered by 95% of the dose (V95% > 95%) and to avoid hot spots > 105% for treatments prescribed to mean dose according to International Commission on Radiation Units and Measurements (ICRU) 83 report [31].

For treatments normalized to a specific isodose the plan objectives were to have 100% of PTV covered by prescription isodose and to avoid hot spots > 140% according ICRU 91 report [32].

The minimal set of dose volume constraints used for OARs optimization were: for rectum, V10 Gy < 40%, V18 Gy < 20%; for bladder, V10 Gy < 25%, V18 Gy < 15%; for femoral heads, V24 < 10%; for penile bulb, V24 < 50%; for small intestine, V18 < 5 cm3 and for urethra Dmax value < 120% of prescribed dose, according to current evidences [33, 34].

Clinical evaluations were performed during the treatment, 30 days after RT course completion and then every three months.

Acute and late toxicities were assessed using Common Terminology Criteria for Adverse Events (CTCAE) v4.0 and v5.0 [35, 36].

Toxicity incidence, local control (LC), distant progression free survival (DPFS), biochemical recurrence free survival (BRFS) and overall survival (OS) rates were registered. Actuarial outcomes results were analyzed through the Kaplan-Meier method; log-rank tests were used to evaluate subgroups differences. Prism version 8.31 for macOS software (1994–2019 GraphPad Software, La Jolla California USA, www.graphpad.com) was used to perform statistical analyses.

## Results

Data from 18 patients who underwent MRgSBRT re-treatment for prostate or prostate bed recurrences were collected. The patients were treated in five different institutions: 6 patients were enrolled at Fondazione Policlinico Universitario “A. Gemelli” IRCCS in Rome; 5 patients were enrolled at Ospedale San Pietro Fatebenefratelli in Rome, Italy and University Hospital of Munich (LMU), while both Mater Olbia Hospital and Heidelberg University Hospital enrolled 1 patient.

The median time between EBRT course and relapse was 10 years (range 5–17), while median age at recurrence time was 77 years (range 60–86).

In 9 patients (50%) relapse occurred after adjuvant treatment following radical prostatectomy, while intraprostatic relapse occurred in 9 patients (50%).

Reirradiation consisted of a MRgSBRT treatment delivered with a target mean normalization according to ICRU 83 report in 6 patients (33.3%) or isodose normalization between 80 and 86% according ICRU 91 report in 12 patients (66.7%).

A nominal dose less or equal than 30 Gy was prescribed in 6 patients (33.3%) while a dose higher than 30 Gy was prescribed in 12 patients (66.7%). Median biologically effective dose (BED) of MRgSBRT re-treatment was 213,3 Gy (103,1-560), considering an α/β of 1.5.

All patients were treated on alternate days with a median number of fractions of 5 (range 4–6); in 5 patients (27.8%) an online adaptive treatment was performed when deemed necessary by the treating physician.

OARs dose constraints and delivered doses are reported in Table 2.

**Table 2:** OARs dose costraints

OAR	Dose constraint	Recommendation	Median value	Range
Rectum	V10	< 40%	24.71%	0-45.9
	V18	< 20%	3.04%	0-12.70
Bladder	V10	< 25%	15.32%	0-78.6
	V18	< 15%	4.62%	0,5–43,41
Femoral heads	V24	< 10%	0%	0
Penile bulb	V24	< 50%	0%	0–8,9
Small bowel	V18	< 5 cm^3^	0%	0-6.42
Urethra	Dmax	< 120% of prescribed dose	86.10%	2.18–108.00

**Table 3:** 1-year and 2-years percentage for investigated clinical outcomes

Outcome	1 year	2 years
OS	88.9%	88.9%
LC	88.9%	66.7%
DPFS	53.1%	53.1%
BRFS	88.9%	66.7%

OS: overall survival; LC: local control; DPFS: disease progression free survival; BRFS: biochemical relapse free survival

Genitourinary (GU) acute Grade 1 toxicity events occurred in 2 patients (11.1%); 4 patients suffered from gastrointestinal (GI) toxicity with Grade 1 and Grade 2 events in 1 (5.6%) and 3 (16.8%) patients, respectively. Multivariate analysis showed no correlation between any grade toxicity events and dose prescription such as for OARs dose values exceeding suggested constraints values. All patients completed the foreseen treatment schedule without interruptions.

Median follow-up time was 4 months (range 1–39) and late toxicity assessment was performed in 13 patients (83.3%).

Late Grade 1 GU toxicity was seen in 3 patients (16.8%), no Grade 2 or greater GU toxicity occurred; 3 (16.8%) patients showed Grade 1 late GI toxicity while Grade 2 event was observed in 1 (5.6%) patient.

1-year and 2-years LC rates of 88.9% and 66.7%, respectively, with 16 patients (88.9%) being free from local failure in the considered time frame. Complete response was observed in 4 patients (22.2%), while partial response and stable disease were observed in 4 patients (22.2%) and 8 patients (44.4%), according to RECIST version 1.1 criteria [37].

Following reirradiation, 2 patients (11.1%) underwent local failure at 4 and 24 months, respectively, both in the prostate bed. No significant differences were found at dose prescription subgroup analysis. BRFS occurred in 2 patients (11.1%) with a 1-year rate of 69.64% and a median PSA value of 0.8 ng/ml (range < 0.001–131), both patients with biochemical failure showed also distant failure.

Distant progression was shown in 5 patients (27.8%) with 1-year and 2-years DPFS rates of 53.1%, no significant correlation to prescribed dose was shown in subgroup analysis. Only 1 patient (5.6%) died of non-cancer related event (sepsis) five months after reirradiation. The 1-year and 2-years percentage for investigated clinical outcomes are summarised in Table 3.

## Discussion

This multicentric experience represents a preliminary analysis in terms of feasibility and efficacy of MRgSBRT reirradiation in a cohort of 18 previously irradiated patients with PC local relapse, using a 0.35 T hybrid LINAC. The low incidence of high-grade toxicity and the good results in terms of efficacy, although related to a modest number of patients, would seem to encourage the use of this technique.

The occurrence of PC relapse after local treatments is still relevant despite the increasing accuracy and technological advancement of multimodal treatments. The new imaging modalities also provide high accuracy for local relapse diagnosis, also in absence of needle biopsy [38, 39].

The best treatment strategy in this setting is still unclear and no consensus has been reached about the best approach, especially considering the availability of different local therapy options such as salvage cryotherapy, high-intensity focused ultrasound ablation (HIFU), HDR brachytherapy, and salvage radical prostatectomy [11–16, 40].

Regardless these alternatives, observation and androgen-deprivation therapy have historically been the preferred option and the number of patients treated with local salvage therapies still remains low, despite the curative potential of this approach and the advantage of postponing the eventual use of systemic therapies [10, 41].

The use of RT in this setting could represent a valid alternative to surgery, which is an approach not devoid of sequelae impacting on the quality of life of the patient, such as urinary incontinence, impotence and anorectal dysfunction [12, 14, 42].

Considering the implementation of modern radiotherapy modalities reirradiation could be considered as a feasible and safe approach [17, 43–47].

Interesting preliminary results suggest that more than 50% of patients who have undergone salvage reirradiation are biochemically relapse-free with very low rates of severe toxicity [48].

The role of HDR-BT has been widely assessed, recent systematic reviews considering the evidence reported in literature has showed good results in terms of toxicity and oncological outcomes [17, 49]. HDR-BT showed promising results in terms of biochemical control in different experiences with a 2-year median BRFS of 74%, with limited occurrence of G3 toxicity.

Literature data showed that SBRT represents the second therapeutic strategy used in local re-treatments, after brachytherapy. In particular image-guided radiotherapy, combined with HDR-BT and SBRT, allows a higher sparing of the surrounding OARs, with a sharper gradient of dose, while still maintaining ablative dose [16, 50].

The optimal dose prescription for prostate reirradiation is not yet standardized. Corkum et al. recently performed a meta-analysis in order to describe the oncologic and toxicity outcomes for salvage reirradiation with EBRT and SBRT with a median reirradiation dose in EQD2 of 77.1 Gy (α/β = 1.5), with 92% patients receiving SBRT [51]. The authors observed an increase in local control and biochemical relapse free survival for higher EQD2 doses, even though high rates of GU and GI toxicities occurred. Partial prostate re-RT appears promising as it showed the decrease of toxicity with no apparent negative impact on disease control outcomes [51].

Precise knowledge on long-term recovery of occult radiation injury in various OARs is essential and literature data showed that accepted cumulative reirradiation dose should not exceed 120 Gy for bladder and 70–100 Gy for rectum [52, 53].

The adoption of a hydrogel rectal spacer could be a possible strategy to reduce the dose to healthy organs. Hamstra et al. performed a single-blind phase III trial of IGRT-IMRT with the adoption of a device called SpaceOAR, a FDA–approved hydrogel intended to create a rectal–prostate space, providing strong evidence for the benefit of its use in prostate irradiation in terms of rectal and urinary morbidity [54].

Within our population this solution was adopted only in two patients (11.1%), with good tolerance during RT sessions and without substantial differences in terms of GI toxicity compared with those who did not use it.

Other strategies to improve the reproducibility of patient positioning, such as the supine over prone set-up position, endorectal balloons, bladder-filling protocols and bladder ultrasound image guided radiation therapy (IGRT) have also been used in order to standardize organs’ volume and consequently manage internal organ motion during pelvic irradiation, although their clinical benefit still remains uncertain [55].

Historically, cone beam computed tomography (CBCT) has been largely employed during RT and SBRT treatment in order to ensure correct patient positioning, provide anatomical information during RT and to overcome the uncertainties arising from organ motion, but soft tissues are challenging to locate with standard CBCT-image-guidance techniques.

Bladder and rectum also suffer from inter-fraction volume variation with potentially significant effects on the cumulative dose received: while volumetric dose received by the bladder decreases as its volume increases, the inverse effect was observed for the rectum [56]. The robotic Cyberknife technique with the use of fiducial marker implantation has also been shown to be a safe approach for reirradiation, assuring a high accuracy of target positioning [22, 34].A recent experience on 64 patients treated with cyberknife modality showed a 2-year LC rate of were 75%, with 1-year LC rate for BED ≥ 130 Gy 85% [22]. The analysis of toxicity profile demonstrated the safety of the procedure as only 1 patient showed grade 3 late GU toxicity.

The availability of MR-hybrid RT devices has allowed the introduction of MR-guided IGRT that can overcome the uncertainties associated with x-rays based IGRT [25, 27]. MR-guidance provides excellent visualization of soft tissues, especially for lung, pelvic and abdominal neoplasms [24–27].

During treatment delivery a cine-MRI is acquired in a sagittal plane in in order to automatically gate treatment beams. The contours of target volume and a boundary structure are automatically deformed and transferred onto different cine MR frames. During the treatment session, the beam automatically shuts off, if a user-defined percentage of the volume of interest is outside of the user-defined boundary [57]. This method has increased delivery precision during both free-breathing and gated treatments with decreased toxicity for close tissues [58]. Interim analysis of MIRAGE phase III randomized trial have also shown a statistically significant reduction in acute grade ≥ 2 GU toxicity with MRI-guidance versus CT-guidance in the context of prostate SBRT [59].

The implementation of MRgSBRT in the PC reirradiation has also been hypothesized by other groups, who described encouraging results in terms of feasibility, toxicity reduction and clinical outcomes [29, 30].

Our experience reports the preliminary results in terms of MRgSBRT PC-reirradiation performed with 0.35 T hybrid units in five different institutions.

MRI sequences can reduce the daily uncertainties in identifying the exact interface between the posterior part of the prostate gland and the anterior rectal wall or between the prostate apex and the penile bulb, allowing a better definition of the daily critical structures and consequently the possible reduction of PTV margins [60, 61].

Our results are in line with those already reported in the literature in terms of local control, as in our cohort a 1-year control rate of 88.9% was shown [62].

As for acute toxicity rates, only grade 1 GU toxicities events were observed in our cohort, and only 3 patients (16.7%) suffered from grade 2 GI toxicities, which are comparable results to the published data [7–56].

Salvage SBRT reirradiation for locally recurrent PC offers a satisfactory tumor control: an Italian mono-institutional study on 64 patients demonstrated 1-year biochemical progression-free survival rate and clinical progression-free survival rate of 85 and 90%, respectively, with excellent toxicity profile [22].

Our results are also comparable to recent experience of PC reirradiation by means of MRgSBRT. Alongi et al. reported in a preliminary report of 22 patients grade 2 GI and GU acute toxicity events in 4 patients (18%) and a BRFS 1-year rate of 85.9% [29].

Similarly, Michalet et al. showed low rates of acute grade 1 GI toxicity rates with no grade 2 events and no grade 2 GI acute toxicity events at 3 months FUP. In the cohort of 37 patients at 1 year follow up, the BRFS rate was 65% [30].

SBRT treatments delivered with MRgRT have been investigated for abdominal and pelvic neoplasms, supporting the opportunity to perform online treatment plan adaptation, by optimizing the dose distribution on a daily basis under MRI guidance with reduced PTV margins [24–27].

Henke et al. described the first experience of stereotactic adaptive MRgSBRT in the upper abdominal malignancies’ scenario, demonstrating that this approach is feasible and safe, and also allows to perform a dose escalation and simultaneous OARs sparing. [63]

Finally, it should be noted that this study has some limitations. First, the limited number of patients, mainly related to the fact that the number of centers equipped with MRgRT units is still very low. Secondly the heterogeneous range of prescription doses and the limited observation period could represent biases that should be considered in defining the correlation with clinical outcomes and toxicity.

Nevertheless, we can state that MRgSBRT seems to offer the opportunity to overcome the traditional limitations of prostatic reirradiation, ensuring precise delivery of the high doses and OARs sparing through an accurate target visualization and the application of cutting-edge gating and online adaptive replanning protocols.

Longer follow-up and a larger number of patients are necessary to evaluate reirradiation effectiveness and optimal patient selection criteria in order to identify the population most suitable for this innovative and promising therapeutic approach.

## Data Availability

Datasets used and analyzed for this study could be provided upon reasonable request from corresponding author.
